# Lipid emulsion-induced hypertension post resection of pediatric neuroblastoma: a case report and literature review

**DOI:** 10.1186/s12887-022-03278-2

**Published:** 2022-04-25

**Authors:** Lihua Yuan, Tao Li, Lijuan Yuan, Feng Chen, Jinchun Qiu, Xing Ji

**Affiliations:** 1 Department of Pharmacy, Children’s Hospital of Nanjing Medical University, 210008 Nanjing, China; 2 Department of Oncology, Children’s Hospital of Nanjing Medical University, 210008 Nanjing, China; 3 Department of Gynecology, Yancheng Maternity and Child Health Care Hospital, Yancheng, 224002 China

**Keywords:** Parenteral nutrition, Lipid emulsion, Adverse reactions, Hypertension

## Abstract

**Background:**

Parenteral Nutrition (PN) is preferred when patient is unable to eat. Most clinically widely used lipid emulsion is now attracting more attention in its stability and adverse reactions. We report here the first case of lipid emulsions caused hypertension.

**Case presentation:**

A 1.5 years old girl was diagnosed with neuroblastoma and underwent chemotherapy subsequently followed by resection surgery. She received PN for nutritional support after surgery. with the initiation of PN, this patient developed hypertension. Possible causes of hypertension were evaluated. After the discontinuation of lipid emulsions in PN, her hypertensive symptoms ceased. The lipid emulsion was therefore considered as the cause of her hypertension.

**Conclusions:**

The pathogenesis of hypertension caused by fatty milk is possibly associated with increased production of reactive oxygen species, increased oxidative stress and vasoconstriction.

## Background

Parenteral nutrition (PN) is a nutritional support when a patient's gastrointestinal tract is either unavailable or unreliable for more than 5 to 7 days or when extended bowel rest is desired with therapeutic reasons [1]. Lipid emulsion is considered to be essential part of clinical PN applied to provide fatty acids and energy. Since the 1960s, new concepts in the field of nutritional support therapy have been continuously improved and updated. Lipid emulsion is now attracting more attention in its stability and adverse reactions [2].

Adverse reactions of lipid emulsions include anaphylactic shock, high fever, arrhythmia, and fat overload syndrome, etc. [3]. To the best of our knowledge, there is not any report on the relationship between lipid emulsions and blood pressure fluctuations.

Here, we reported the first case of lipid emulsions induced hypertension in a female toddler after neuroblastoma surgery.

### Case presentation

**Fig. 1:**
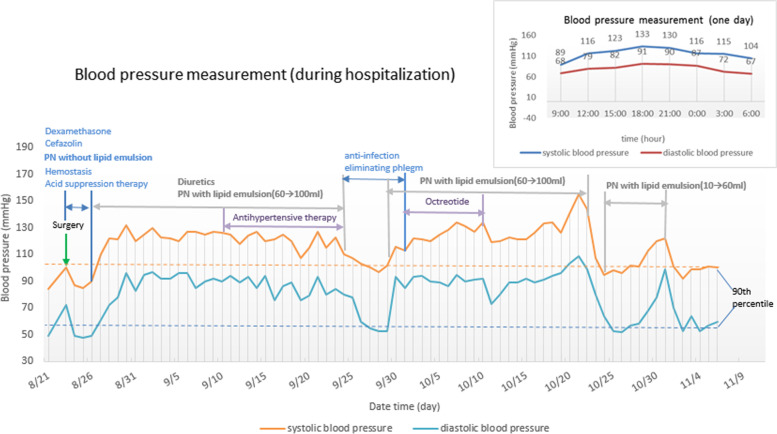
Blood pressure measurement. Changes in blood pressure during the period of therapy, including the relationship between blood pressure changes and all relevant drugs. The 24-h blood pressure monitoring (on October 17th) is shown in the right upper corner of the figure

On August 23rd (day 3 of hospitalization) 2021, a 1.5-year-old girl underwent abdominal mass resection surgery after chemotherapy for neuroblastoma. Before surgery, the physical examination revealed that the patient weighed 10.0 kg, with a height of 82.3 cm. Level of systolic and diastolic blood pressure within the normal range (as shown in Fig. 1). A transitory increase in blood pressure (BP) is observed following surgery, with a blood pressure of 101/57 mmHg, higher than the 90th percentile (100/56 mmHg) and less than 95th percentile (103/60 mmHg) for 1-year girl [4]. After the operation, the child was given antibiotics, hemostasis, acid suppression therapy, and nutritional support. On August 30th, Facial Pain Scale (FPS) scores ≤ 2 was best described for child with postoperative pain as mild pain.

PN without lipid emulsion was administrated after surgery (on August 24th) with continuous infusion for about 20 h. Dextrose, amino-acids, electrolytes, vitamins, and trace elements were mixed in a well-defined order to prepare total nutritional admixture. Before starting nutrition therapy, levels of total cholesterol (TC), triglycerides (TG), low density lipoprotein cholesterol (LDL-C) and high-density lipoprotein cholesterol (HDL-C) were tested and were within the normal range. On August 27th, 20% medium-chain triglycerides / soybean oil-based lipid emulsion was added to the PN solution, at a starting dose of 0.5 g/kg/day, gradually increased to 100 ml (2.5 g/kg/day). However, the blood pressure were increased to 125/95 mmHg. According to cardiology consultation, diuretic and benazepril hydrochloride were ordered, and the patient’s blood pressure tended to become stable gradually. During the therapy, the levels of serum lipids were normal. For total fluid output at 24 h after admission, urine output, abdominal drainage and stool, were all measured. then, as measures of fluid balance, we calculated the total fluid intake at 24 h after admission.

On September 25th, she suddenly developed a fever with shivering chills. The laboratory test demonstrated a high neutrophil count (4200/μL) and low white blood cell count (4300/μL). To ensure the effectiveness, preemptive treatment with i.v. ceftazidime (100 mg/kg/d) and vancomycin (40 mg/kg/day) were administered. Later that day she experienced a fever to 40.7 °C, hypotension, tachycardia and septic shock. Blood and peripherally inserted central catheter (PICC) tip cultures grew Staphylococcus aureus. The patient was given broad-spectrum antibiotics and the PICC was removed. The patient’s condition was improved quickly. PN and antihypertensive agents were withdrawn during the period of therapy for PICC-related bloodstream infection. More importantly, the blood pressure level remained within the normotensive range. After infection symptoms getting better, the nutritional support was resumed because of the requirement for maintaining or improving the immune competence (the patient developed chylous ascites). However, once the PN was resumed, with lipid emulsion at dose of 100 ml (2.5 g/kg/day), the blood pressure began to fluctuate again, usually ranging from 120/90 to 134/95 mmHg, with the highest as 155/109 mmHg.

After consultation with clinical pharmacist, the PN was discontinued, and blood pressure subsequently decreased to 107/80 mmHg (on October 23rd). The blood pressure monitoring results showed that the patient’s high blood pressure was likely to be caused by the PN mixture or a certain component in the PN.

For the next two days, the PN without lipid emulsion was administrated as recommended by the clinical pharmacist. The patient's blood pressure remained stable (95/64 mmHg). We therefore conclude that the blood pressure fluctuations were associated with the lipid emulsion in the PN. However, considering the need for nutritional support of long-term treatment in children with chylous ascites, clinical pharmacist recommended starting with low-dose lipid emulsion and increased by 10 mL (0.25 g/kg/day) each time. However, when the volume of lipid emulsion in the PN was increased to 60 mL (1.5 g/kg/day), the blood pressure began to fluctuate from 120/78 to 122/99 mmHg. Tailoring the dose of lipid infusion at 45 mL, the patient could maintain stable blood pressure (92/53 to 101/70 mmHg). With continued such treatment, she eventually discharged from the hospital.

## Discussion and conclusions

Since the 1960s, new concepts in the field of nutritional support therapy have been continuously developed and updated. Lipid emulsion is receiving much attention in its stability and adverse reactions [2, 3]. New uncommon adverse reactions are being constantly reported, such as fat overload syndrome caused by lipid emulsions, characterized by high triglycerides, fever, hepatosplenomegaly, coagulopathy, and organ failure [5, 6]. To the best of our knowledge, this is the first case of adverse reaction pertaining hypertension caused by the lipid emulsions in the PN solutions.

**Fig. 2:**
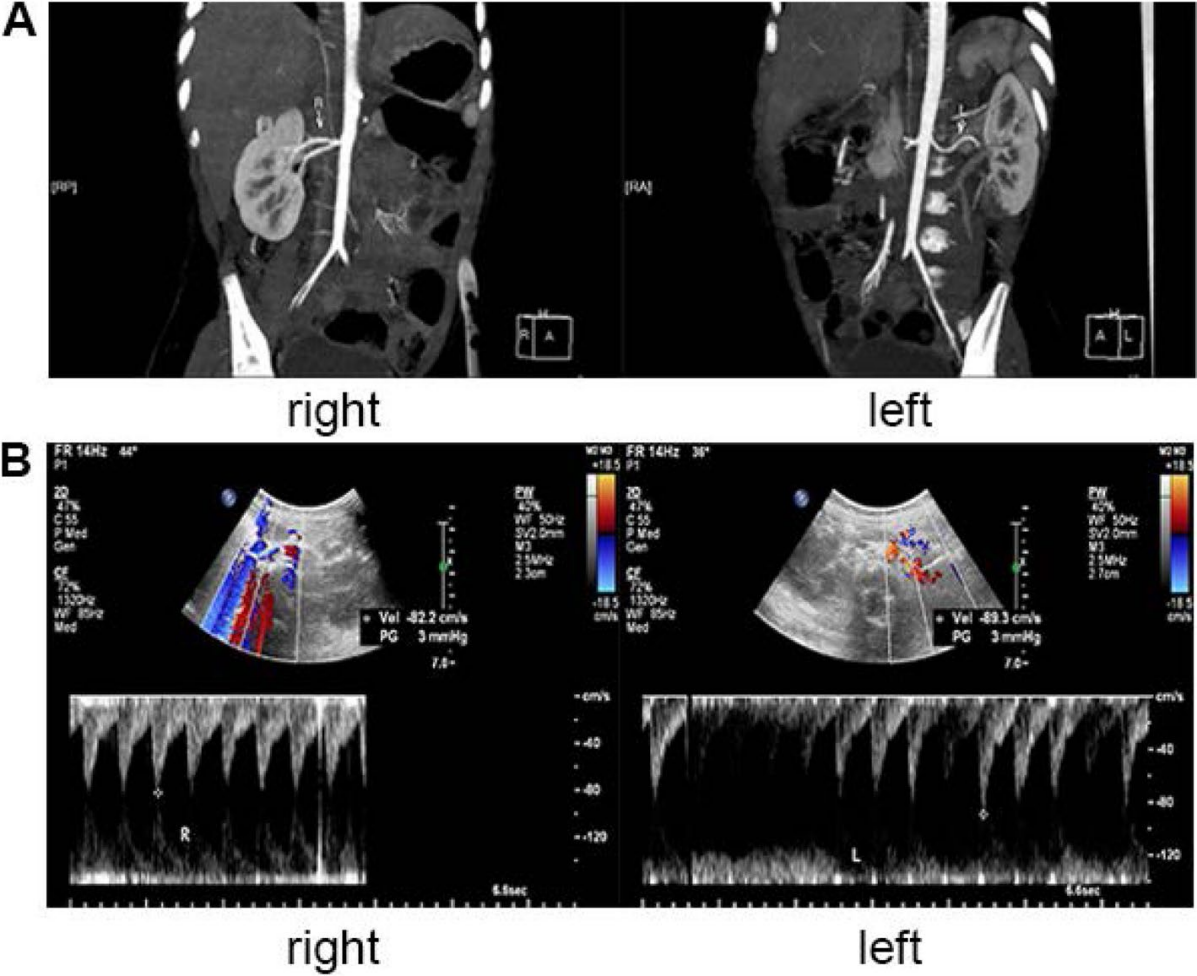
Contrast-enhanced computed tomography of the abdomen and renal artery Doppler ultrasound. **a** contrast-enhanced computed tomography of one-and-a-half-year-old girl: the abdomen image shows normal renal arterial (white arrow); **b** Renal artery Doppler ultrasound: the internal diameter for the right renal artery was 2.2 mm at the beginning, the maximum forward blood flow velocity was 89.3 cm/s (in normal range: 69.61 ± 17.77 cm/s); the internal diameter for the left renal artery was 2.5 mm at the beginning, the maximum forward blood flow velocity was 82.2 cm/s (in normal range: 69.61 ± 17.77 cm/s)

In this case, the child's vital signs have been improved remarkably after therapy. However, the patient's blood pressure increased significantly after the nutritional medication as an add-on treatment for chylous peritonitis. Moreover, 24-h blood pressure monitoring revealed that blood pressure fluctuations were related to nutrient solutions (Fig. 1). The observation worthy of mention is that the patient's blood pressure did not rise after only using the PN solution without lipid emulsion, and it is important to explain why we consider the lipid emulsion responsible for her hypertension. In addition, contrast-enhanced computed tomography of the abdomen and renal artery Doppler ultrasound showed that the normal renal arterial (Fig. 2), and there were no symptoms of the nervous system at any stage of infection. Therefore, we concluded that PN emulsion could lead to the hypertension symptom.

The Naranjo method, an algorithm to assess the likelihood of an adverse drug reaction, was used to determine whether the lipid emulsion was the cause of hypertension [7, 8]. The Naranjo method yielded a probability score of + 10, suggesting that the lipid emulsion was the perpetrator of the adverse hypertension (Table 1). The consultation of clinical pharmacist was proved to be correct. The pathogenesis of hypertension caused by lipid emulsion remains unknown. It might be associated with increased production of reactive oxygen species, resulting in increased oxidative stress and vasoconstriction [9]. It might also be associated with decreased NO bioavailability through inhibition or scavenging of NO release [10].

In conclusion, delayed identification of the adverse hypertension probably gives rise to many unfavorable consequences, such as unnecessary and costly laboratory examinations, misuse of antihypertensive drugs, especially the possible occurrence of more adverse reactions. Further researches were warranted to confirm the correlation between hypertension and fat emulsion.

**Table 1:** Naranjo’s scale for likelihood of hypertension caused by lipid emulsion

Naranjo’s scale				
Question	**Yes**	**No**	**Don’t know**	**Score for hypertension**
**1. Are there previous conclusive reports on this reaction?**	+ 1	0	0	0
**2. Did the adverse event appear after the suspect drug was administered?**	+ 2	-1	0	+ 2
**3. Did the adverse reaction improve when the drug was discontinued or a specific antagonist was administered?**	+ 1	0	0	+ 1
**4. Did the adverse reaction reappear when the drug was re-administered?**	+ 2	-1	0	+ 2
**5. Are there alternate causes (other than the drug) that could have solely caused the reaction?**	-1	+ 2	0	+ 2
**6. Did the reaction reappear when a placebo was given?**	-1	+ 1	0	+1
**7. Was the drug detected in the blood (or other fluids)in a concentration known to be toxic?**	+ 1	0	0	0
**8. Was the reaction more severe when the dose was increased or less severe when the dose was decreased?**	+ 1	0	0	+ 1
**9. Did the patient have a similar reaction to the same or similar drugs in any previous exposure?**	+ 1	0	0	0
**10. Was the adverse event confirmed by objective evidence?**	+ 1	0	0	+ 1
Total				+ 10

Scoring for Naranjo’s algorithm; ≥ 9 = definite; 5–8 = probable; 1–4 = possible; 0 = doubtful

## Data Availability

The datasets used and/or analyzed during the current study are available from the corresponding author on reasonable request.

## Abbreviations

PN: Parenteral nutrition
TC: Total cholesterol
TG: Triglycerides
LDL-C: Low density lipoprotein cholesterol
HDL-C: High-density lipoprotein cholesterol
PICC: Peripherally inserted central catheter
